# Review in Structure–Activity Relationships, Synthetic Regulation, and Applications of Mono/Di-Rhamnolipids

**DOI:** 10.3390/microorganisms14030570

**Published:** 2026-03-02

**Authors:** Zhen Li, Liuyu Gao, Shengze Su, Rui Wang, Peng Lei, Yian Gu, Yongting Song, Hong Xu, Liang Sun

**Affiliations:** 1 College of Food Science and Light Industry, Nanjing Tech University, Nanjing 211816, China; lzzzzz@njtech.edu.cn (Z.L.); passerby@njtech.edu.cn (L.G.); 202461119010@njtech.edu.cn (S.S.); ruiwang2013@njtech.edu.cn (R.W.); lei-peng@njtech.edu.cn (P.L.); yian.gu@hotmail.com (Y.G.); xuh@njtech.edu.cn (H.X.); 2 Research Institute of Petroleum Engineering and Technology, Sinopec Shengli Oilfield, Dongying 257000, China; songyongting.slyt@sinopec.com

**Keywords:** biosurfactants, mono/di-rhamnolipids, surface activity, bioactivity

## Abstract

Rhamnolipids (RLs) are natural biosurfactants produced by microbial fermentation. They exhibit excellent surface activity, environmental compatibility, and versatile biological activities, with broad application potential in energy development, environmental remediation, personal care, and biomedicine. In recent years, RLs have garnered significant attention due to the structural divergence between mono-rhamnolipids (mono-RLs) and di-rhamnolipids (di-RLs). Their distinct physicochemical and biological properties necessitate structure-function-guided “tailor-made” synthesis. Notably, the compositional fluctuation of naturally occurring RL mixtures constrains their high-value utilization. This review systematically summarizes the structure–function relationships, microbial biosynthetic pathways, and regulatory approaches of mono-RLs and di-RLs, with a core focus on the targeted production of RLs with specific mono-/di-RL ratios. The review also highlights the application-specific suitability of the two RL congeners. It aims to provide a theoretical foundation and technical reference for the precision manufacturing and commercialization of RLs in energy, environment, health, and other related fields.

## 1. Introduction

Biosurfactants are natural amphiphilic molecules synthesized by microorganisms, featuring efficient surface activity, environmental friendliness, and diverse biological properties, which endow them with broad application prospects in energy, environmental remediation, and biomedicine [1]. Among them, rhamnolipids (RLs) are the most extensively studied glycolipid biosurfactants with the strongest commercial potential, becoming a core research focus in this field [2]. Guided by green chemical engineering and sustainable development concepts, RLs outperform traditional synthetic surfactants due to their biodegradability, low ecotoxicity, and functional adjustability derived from structural diversity [3]. Their application scope has expanded from traditional fields like microbial enhanced oil recovery and contaminated site remediation to high-value-added areas such as personal care and pharmaceutical carriers, showing enormous market potential [4].

**Figure 1:**
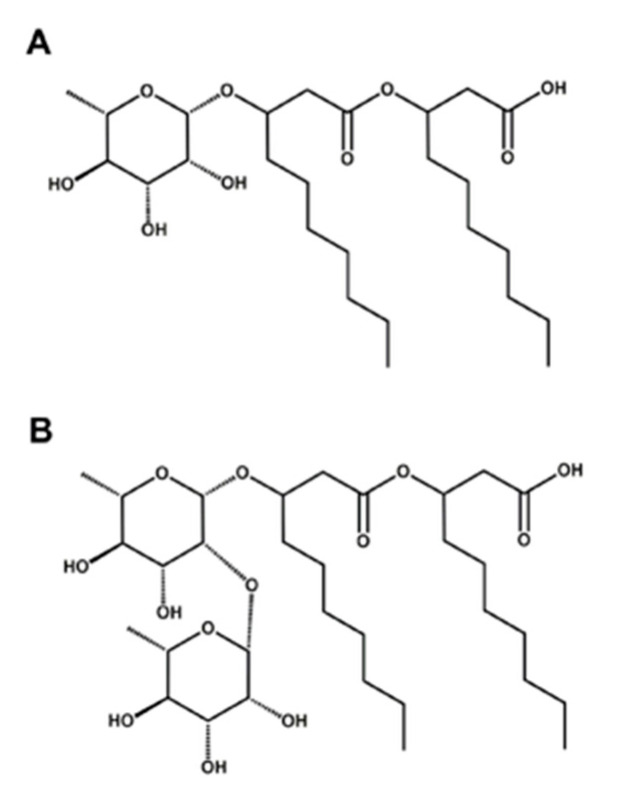
General molecular formula of RLs. (**A**) Mono-RLs. (**B**) Di-RLs.

Since Jarvis and Johnson first isolated RLs from *Pseudomonas aeruginosa* in 1949 [5], over 60 structural variants have been identified [6]. All RLs are composed of 1–2 hydrophilic rhamnose moieties covalently linked to 1–2 hydrophobic β-hydroxy fatty acid chains through glycosidic linkages, with two distinct structural subtypes identified as mono-rhamnolipids (mono-RLs) and di-rhamnolipids (di-RLs) (Figure 1). RLs have excellent surface activity, which can reduce the surface tension of water from 72 mN/m to 30–32 mN/m [7], and their hydrophile–lipophile balance (HLB) value typically ranges from 9 to 16, making them efficient oil-in-water (O/W) emulsifiers with good solubilization capacity [8]. Thus, they are widely used in cosmetic formulations and agricultural fertilizer synergists [9]. Additionally, RLs exhibit good salt tolerance and thermal stability, supporting their application in complex environments [10]. These physicochemical properties are closely related to their molecular structures, prompting extensive research on structure–activity relationships.

Currently, research on RL structure–activity relationships mainly focuses on the number of rhamnose groups in the hydrophilic head, specifically, comparing mono-RLs and di-RLs. This core structural difference directly and profoundly affects their physicochemical properties and biological functions, thereby determining their suitable application scenarios [11]. Studies have shown that mono-RLs generally have a lower critical micelle concentration (CMC) and a stronger surface tension reduction ability [12], while di-RLs exhibit enhanced hydrophilicity due to the additional rhamnose group, showing better performance in emulsification stability and foaming properties [13]. However, a key bottleneck limiting their high-value applications is that RLs produced by the fermentation of natural wild-type or engineered strains are usually complex mixtures of multiple congeners [14]. This inherent compositional variability leads to inconsistent batch performance, making it difficult to meet the strict requirements for raw material uniformity and functional precision in fields such as medicine and high-end cosmetics [15]. To address this issue, the strategy of “tailor-made synthesis” or “targeted biosynthesis” has emerged in recent years. By means of rational genetic engineering design and precise fermentation process control, this strategy aims to achieve the targeted, high-yield production of RLs with specific structures, especially controlling the ratio of mono-RLs to di-RLs, so as to meet the precise needs of different downstream applications [16].

Given this context, this review systematically summarizes the differences between mono-RLs and di-RLs in chemical structure, physicochemical properties, biological activities, and synthesis pathways, focusing on analyzing their intrinsic structure–function relationships. It details the differences in their surface activity, emulsification, self-assembly, solubilization and other behaviors, summarizes and discusses targeted regulatory methods based on synthetic biology and metabolic engineering technologies, and generalizes their differentiated applicability in drug delivery, microbial enhanced oil recovery (MEOR), organic pollution remediation, and other fields. The purpose of this review is to provide a solid theoretical foundation and cutting-edge technical perspective for the molecular design, controllable production, and precision commercial application of RLs.

## 2. Differences in the Properties of RLs

### 2.1. Surface Tension and CMC

RLs have a low CMC and excellent surface activity. Studies have shown that the CMC of RLs is regulated by multiple factors, mainly including solution pH, molecular structure, especially the number of rhamnose groups, and salt ion strength [17]. Among these, molecular structure has a particularly critical impact; for example, Müller et al. [11] clearly demonstrated that the number of rhamnose units in the hydrophilic head is the core structural factor determining the CMC. Mono-RLs have a lower CMC (about 25 mg/L), while di-RLs have a higher CMC (about 120 mg/L). This approximately fourfold difference directly reflects the fundamental impact of the relative ratio of hydrophobic chains to hydrophilic heads on molecular self-assembly tendency. Mono-RLs are relatively more hydrophobic and thus undergo self-assembly to form micelles at lower concentrations.

In addition to structure, environmental pH is another key factor that dynamically regulates the CMC of RLs by altering molecular ionization states. Shin’s [18] study on the relationship between the RLs’ CMC and pH showed that when the pH < 7.0, CMC gradually decreased with increasing pH, reaching a minimum around pH 7.0; when pH > 7.0, CMC gradually increased with rising pH. This non-linear variation arises from the delicate balance between hydrogen bonding and electrostatic repulsion. In the acidic to neutral range, the carboxyl groups in RL molecules are highly protonated and intermolecular hydrogen bonding networks dominate, which effectively offsets partial electrostatic repulsion and promotes the stable formation of micelles. As alkalinity increases, carboxyl groups are deprotonated and negatively charged, significantly enhancing electrostatic repulsion between the hydrophilic heads, which hinders the micellization process and leads to a rebound in CMC [19]. This mechanism also indirectly confirms that di-RLs, with larger hydrophilic heads, carry more charges, and their micellization process is more sensitive to the electrostatic environment changes caused by pH variations, showing a more obvious pH dependence than mono-RLs.

### 2.2. HLB and Emulsifying Activity

Hydrophilic–lipophilic balance (HLB) is a key physicochemical parameter that quantitatively characterizes the relative strength of hydrophilicity and lipophilicity in surfactant molecules. Its value typically ranges from 0 to 20, with higher values indicating stronger overall hydrophilicity of the molecule. The HLB value directly determines the functional orientation of surfactants in a system and serves as a core theoretical basis for predicting and selecting their suitability for specific scenarios such as emulsification, wetting, solubilization, and cleaning [20]. The HLB value of RLs varies with structural differences such as mono/di-rhamnose type and fatty acid chain length. Among them, di-RLs typically have a higher HLB value (about 16) and strong hydrophilicity, which enables them to more effectively reduce the surface tension of the aqueous phase and exhibit excellent oil-in-water (O/W) emulsion formation ability [21]. The resulting emulsions usually have higher stability and better tolerance to temperature changes and salinity fluctuations, making them widely used in cosmetic emulsion preparation and crude oil recovery [22].

In contrast, mono-RLs have a relatively lower HLB value (about 10). Their molecular structure has smaller hydrophilic groups and relatively stronger hydrophobicity, leading to a greater tendency to adsorb and spread at the oil–water interface. Therefore, mono-RLs perform better in stabilizing water-in-oil (W/O) emulsions with good emulsifying capacity, but the formed emulsion droplets are larger and have moderate stability [23]. Based on these properties, mono-RLs are more suitable for dispersion and emulsification processes dominated by continuous oil phases or highly hydrophobic media, such as the petroleum industry [24]. This functional differentiation guided by HLB value differences fundamentally originates from the distinct amphiphilic balances of mono-/di-RLs, which is a key intrinsic property determining their respective optimal application scenarios [24].

### 2.3. Foaming Properties

Foaming properties are a key indicator for evaluating the application potential of surfactants in personal care and cleaning products. Studies have shown that the foaming properties of RLs are closely related to their structure. Tiso et al. [25] reported that di-RLs exhibit a foaming capacity and foam stability comparable to sodium dodecyl sulfate (SDS), and even better performance in hard water, mainly due to their structural tolerance to calcium and magnesium ions. In contrast, mono-RLs have a smaller molecular weight and relatively stronger hydrophobicity, which may give them a higher diffusion coefficient in oil-phase solutions and stronger hydrophobic driving force, enabling faster adsorption to newly formed gas–liquid interfaces. Therefore, mono-RL systems can generate a large amount of foam more quickly in certain dynamic foaming processes such as vigorous stirring and aeration [26]. However, compared with widely used surfactants such as anionic sodium alcohol ether sulfate (AES) and non-ionic alkyl polyglycoside (APG), the foaming capacity of single RL systems is still significantly insufficient, which necessitates a higher dosage in practical applications and thus elevates the overall costs [18,25].

To address this limitation, research has mainly focused on improving the foaming properties of RLs and reducing their dosage through blending strategies. For instance, blending sodium cocamidopropyl sulfate (ACS) with di-RLs can significantly enhance foam stability, which is derived from the synergistic arrangement and intermolecular interactions of the two components at the interface [27]. In addition, the incorporation of silk fibroin (SF), a natural biopolymer, not only significantly improves the foaming capacity and foam stability of di-RLs but also retains their inherent biocompatibility and biological activity, providing a new approach for the development of green formulations with both efficient cleaning and mild properties [28]. Therefore, rational blending design based on intermolecular interactions can not only maximize the green advantages of RLs, such as environmental friendliness and biodegradability, but also effectively compensate for their poor foaming properties, thus remarkably improving their application potential and market competitiveness in high-end daily chemical products.

### 2.4. Aggregation Morphology in Solution

The self-assembly behavior of RLs in aqueous solutions, as well as the morphology and size of the formed aggregates, are highly sensitive to environmental conditions. This sensitivity originates from their amphiphilic molecular structure, especially the charge and spatial characteristics of the hydrophilic groups. Studies have shown that mono-RL morphology is extremely sensitive to environmental factors such as pH and divalent cations [29]. Divalent cations can effectively bridge negatively charged carboxylate groups, significantly reducing the electrostatic repulsion between head groups, thereby strongly inducing vesicle formation or even precipitation. In contrast, di-RLs have larger hydrophilic groups with stronger steric hindrance and electrostatic repulsion, so their aggregate morphology generally exhibits higher tolerance to divalent cations than mono-RLs.

Meanwhile, pH exerts a significant impact on the morphology of RLs. Under acidic conditions (pH ≈ 4.3), RLs tend to form vesicular aggregates; when the pH increases to 6.0–6.5, molecular assembly into lamellar (sheet-like) structures occurs through hydrogen bonding and hydrophobic interactions; in the near-neutral range (pH 6.2–6.6), lipid granular aggregates are additionally observed [30]. As pH further rises to neutral or alkaline conditions (pH > 7.0), the carboxyl groups of RL molecules are fully ionized, and electrostatic repulsion dominates, driving the system to transform into small-sized micelles or even exist as free single molecules. Further studies have shown that the molecular weight of micelles formed by mono-RLs at pH 7.35 is about 38 kDa, while that of micelles formed by di-RLs at pH 8.5 is about 7 kDa, reflecting the significant impact of structural differences on aggregation behavior [31].

In addition, solvent composition can also regulate the aggregation size of RLs. Long et al. [32] found that in the presence of methanol or ethanol, the micelle size of di-RLs decreases with increasing alcohol concentration. When the ethanol concentration is 30% (*v*/*v*) and pH is 9, the hydrodynamic diameter of micelles can even drop below 1 nm. The mechanism lies in alcohol molecules inserting into and interfering with the interactions between the hydrophobic tail chains of RLs, weakening the hydrophobic forces driving micellization, thereby promoting the formation of smaller and more dispersed assemblies. These results indicate that the self-assembly of RLs is a dynamic process synergistically regulated by multiple parameters such as pH, ionic strength, and solvent. Precise “programming” of the morphology and size of their nanoaggregates can be achieved through rational design of environmental conditions. This controllable self-assembly property lays a solid physicochemical foundation for their applications in fields such as drug delivery carriers, nanoreactors, and functional nanomaterials synthesis.

### 2.5. Solubilization

Compared with commonly used synthetic surfactants such as Tween, Triton, and alkylbenzyl sulfonates, RLs exhibit superior solubilization capacity for hydrophobic organic pollutants. They can significantly enhance the solubility of non-aqueous phase liquids, as well as solid or volatile organic compounds such as polycyclic aromatic hydrocarbons and benzene series in the aqueous phase. The solubilization mechanism of RLs mainly relies on micelle formation, where hydrophobic organic pollutants can be encapsulated in the hydrophobic core of micelles for dispersion and “solubilization” in an aqueous environment when their concentration exceeds the CMC. Studies by Arkhipov et al. [33] confirmed that the solubilization of benzene-based compounds can be observed when the RL concentration reaches the CMC, and when the RL concentration increases to 100–200 mg/L, the pollutants can be completely solubilized in the micellar phase. In terms of structural differences, mono-RLs generally have stronger solubilization capacity than di-RLs due to their higher proportion of hydrophobic fatty acid chains. This property can not only be used for organic pollutant recovery and environmental remediation but also promote the microbial degradation of long-chain alkanes. For example, in the presence of mono-RLs, the uptake of hexadecane by *Pseudomonas aeruginosa* ATCC 9027 is significantly enhanced, with a degradation rate exceeding 70% within 16 h [34]. Additionally, the solubilization effect of mono-RLs is also applicable to extracellular polymers in biological systems. Du et al. [35] found in constructed wetland studies that 0.12 g/L of mono-RLs not only promoted microbial growth, but also enhanced the dissolution and dispersion of extracellular polymers, accelerating the release of encapsulated enzymes and thereby improving the overall pollutant removal efficiency of the system. This indicates that the solubilization function of RLs plays multiple roles in environmental biotechnology, demonstrating significant positive regulatory potential in various aspects.

## 3. Biosynthesis of RLs

With the advancement of biosurfactant research, RLs have attracted significant attention due to their excellent surface activity, versatile biological activities, and good environmental compatibility, and their application potential in various fields has become increasingly prominent [36]. Over the past few decades, research on such biosurfactants has continued to grow, and various RL-producing strains have been successively reported [37]. Among genera such as *Pseudomonas* and *Burkholderia*, *P. aeruginosa* exhibits the highest RL production and has been used as a model strain to study the key genes involved in RL biosynthesis [38].

**Figure 2:**
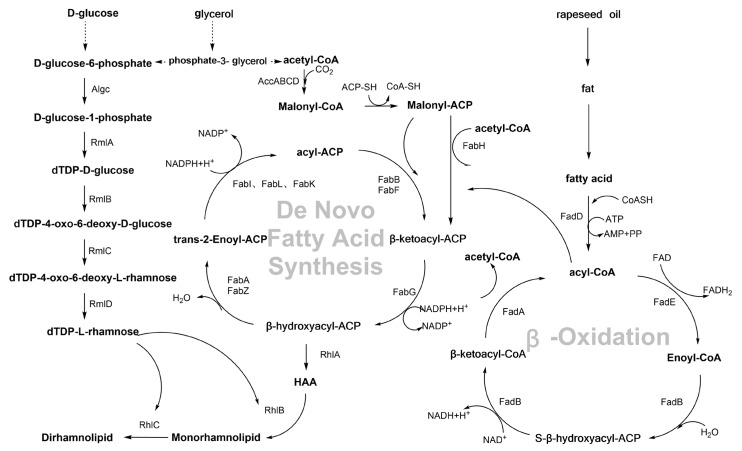
Biosynthetic pathway of RLs. AlgC: Phosphomutase; RmlA: Glucose-1-phosphate thymidylyltransferase; RmlB: dTDP-glucose 4,6-dehydratase; RmlC: dTDP-4-keto-6-deoxyglucose 3,5-epimerase; RmlD: dTDP-4-keto-rhamnose reductase; FabA: β-hydroxyacyl-ACP dehydratase; FabB: β-ketoacyl-ACP synthase I; FabF: β-ketoacyl-ACP synthase II; FabG: β-ketoacyl-ACP reductase; FabH: β-ketoacyl-ACP synthase III; FabI, FabL, FabK: Enoyl-ACP reductase; FabZ: β-hydroxyacyl-ACP dehydratase; AccABCD: Acetyl-CoA carboxylase complex; FadA: 3-ketoacyl-CoA thiolase; FadB: Fatty acid oxidation complex α-subunit; FadD: Acyl-CoA synthetase; FadE: Acyl-CoA dehydrogenase.

The biosynthetic pathway of RLs is a complex and highly regulated process. In 1963, Burger et al. [39] elucidated the metabolic pathway of RL synthesis in *P. aeruginosa* using radioactive labeling. Subsequently, Soberón-Chávez [40] and Charles [41] confirmed that dTDP-L-rhamnose and 3-(3-hydroxyalkanoyloxy) alkanoic acid (HAA) are the synthetic precursors of RLs. With the deepening of research, the RL metabolic synthetic pathway and related key enzymes have been gradually clarified (Figure 2).

The hydrophilic groups of RLs can be synthesized from glucose-6-phosphate. First, glucose-6-phosphate is converted to glucose-1-phosphate by AlgC, and then dTDP-L-rhamnose is synthesized through four sequential steps (activation, dehydration, isomerization, and reduction) under the action of RmlA, RmlB, RmlC, and RmlD [41]. For the biosynthesis of RL hydrophobic groups, studies have shown that it is dominated by the de novo fatty acid synthesis pathway, which produces β-hydroxyacyl-ACP under the action of *RhlG/FabG* [42]. Bazire et al. [43] confirmed through radioisotope labeling experiments that the fatty acid chain composition of RLs remains stable even with changes in carbon sources, strongly supporting that their synthesis is dominated by the highly conserved classical de novo fatty acid synthesis pathway. In 2012, Zhang et al. [44] reported that intermediate products of β-oxidation such as enoyl-CoA can be reverse “recruited” by specific enzymes such as β-ketoacyl-ACP synthase and fed into the de novo fatty acid synthesis pathway, thus providing additional substrates for the synthesis of β-hydroxyacyl-ACP. This finding clarified the metabolic basis for enhanced RL synthesis in bacteria when using complex carbon sources. Overall, the biosynthesis of RL hydrophobic groups takes de novo fatty acid synthesis as the core pathway, with the β-oxidation pathway supplying substrates to the de novo synthesis pathway. This dual-pathway mechanism ensures that bacteria can efficiently synthesize RLs in diverse environments, serving as a key adaptive strategy for their survival and competition [36,45].

**Figure 3:**
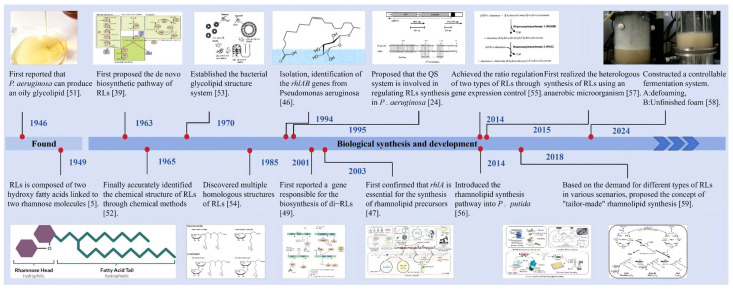
Development process of RLs [5,24,39,46,47,49,51,52,53,54,55,56,57,58,59].

RL synthesis involves multiple steps, and its core pathway consists of a series of highly coordinated enzymatic reactions. With advancing research on RLs, their synthetic pathway and key genes have gradually been uncovered (Figure 3). The *rhlA* gene was first identified by Burger [39] in 1963 as being involved in RL synthesis. The protein RhlA encoded by it plays an important role in the synthesis process, but the specific mechanism was unclear until 1994, when Ochsner et al. [46] found that RhlA may be involved in substrate transport or precursor synthesis through gene mutation and heterologous expression methods. Subsequently, Deziel et al. [47] clarified that *rhlA* is essential for the synthesis of RL precursors: the acyltransferase RhlA encoded by it catalyzes the esterification of two 3-hydroxyacyl molecules to synthesize HAA, a typical precursor of RLs [48]. RhlB, a rhamnosyltransferase I encoded by the *rhlB* gene, is responsible for catalyzing the synthesis of mono-RLs from dTDP-L-rhamnose and HAA. The regulatory genes *rhlR* and *rhlI* downstream of the *rhlAB* operon encode the transcriptional regulator RhlR and synthase RhlI, core components of the quorum sensing system, that regulate the microbial synthesis of RLs. The *rhlC* gene was discovered in 2001, and the rhamnosyltransferase II RhlC encoded by it regulates the synthesis of di-RLs from mono-RLs [49]. *rhlC* is only expressed in the stationary phase after *rhlB* expression is turned off [50]. This coordination and sequence clarifies and enables precise control over the molecular regulation of di-RL synthesis, with the synthesis and content of di-RLs tunable through the upregulation or downregulation of *rhlC* expression following mono-RL synthesis.

## 4. Tailor-Made Synthesis of Mono-/Di-RLs

Mono-RLs and di-RLs exhibit distinct properties such as interfacial activity [11], wettability, and oil-washing efficiency. Adjusting their ratio without changing the total amount allows for adaptation to a broader range of requirements. Based on the demand for different types of RLs in various application scenarios, Wittgens et al. [59] first proposed the concept of “tailor-made RLs”. Subsequently, a growing number of studies have focused on this research direction (Figure 3).

### 4.1. Engineering Modifications for RL Production

After the key genes and enzyme functions in the RL biosynthetic pathway were fully clarified, research entered a phase of regulating the synthetic pathway through genetic engineering modifications. Among these strategies, targeted increase in the proportion of di-RLs by overexpressing or heterologously inserting the *rhlC* gene has become a classic approach. For example, Valdez et al. [60] obtained a functional strain with high di-RL production by expressing the plasmid *prhlAB-R-C* in *P. aeruginosa* ATCC 9027, and the proportion of di-RLs produced by this strain reached 93.6% (Table 1). Similarly, studies by Wu et al. [61] confirmed the effectiveness of this strategy. They heterologously introduced the *rhlC* gene into *P. aeruginosa* SG to construct the engineered strain *P. aeruginosa* SG*rhlC*, and the proportion of di-RLs in the RLs produced by this strain was significantly higher than that of the wild-type strain.

For the targeted production of mono-RLs, the main methods include screening naturally high-yield strains and genetic engineering modifications. For example, *P. aeruginosa* ATCC 9027 is a naturally mono-RL-dominant strain [62]. In addition, blocking the di-RL synthesis pathway by overexpressing the *rhlB* gene or knocking out the *rhlC* gene has become a reliable method to obtain high-purity mono-RLs. For example, Zhao et al. [63,64] constructed the engineered strain *P. aeruginosa* SGΔ*rhlC* by knocking out the *rhlC* gene, and the product of this strain contained 100% mono-RLs. In another study by the same research group [65], knocking out the *rhlC* gene not only achieved the exclusive production of mono-RLs but also increased the total RL yield. Meanwhile, the resulting mono-RL product exhibited good emulsifying activity and oil-washing capacity, expanding its application potential.

**Table 1:** Construction of production strains for different types of RLs.

Strain	Carbon Source	Di-RL Content (%)	Mono-RL Content (%)	Reference
*P. aeruginosa* SG	Peanut Oil	38.54	61.46	[65]
*P. aeruginosa* SG*rhlC*	Glycerol	62.32	37.68	[61]
*P. aeruginosa* SGΔ*rhlC*	Glycerol	0	100	[64]
*P. aeruginosa* ATCC 9027	Rapeseed Oil	0	100	[66]
*P. aeruginosa* ATCC 9027 *prhlAB-R-C*	Rapeseed Oil	93.6	6.4	[60]
*P. aeruginosa* PAO1	Rapeseed Oil	73.3	26.7	
*P. aeruginosa* KT1115	Rapeseed Oil	88.53	11.47	[13]
*P. aeruginosa* LP20	Glycerol	35.5	64.5	[67]
*E. coli* BL21(DE3) DJ208	Glucose	83.9	7.8	[68]
*E. coli* BL21(DE3) DJ208-*fabB*	Glucose	99.3	0.5	
*E. coli* BL21(DE3) DJ208-*fabD*	Glucose	89.6	7.1	
*E. coli* BL21(DE3) DJ208-*fabI*	Glucose	31.6	4.9	
*E. coli* BL21(DE3) DJ208-*fabG*	Glucose	47.4	5.4	

### 4.2. Fermentation Production of Mono-/Di-RLs

RL fermentation faces multiple challenges, including foaming, cost constraints, low yield, and unstable product performance. The most prominent issue is unstable product performance, which can be effectively avoided by producing mono/di-RLs. Under this premise, *P. aeruginosa* ATCC 9027 lacking the *rhlC* gene has become the optimal strain for mono-RL fermentation. In addition to engineering modifications, the selection of fermentation medium and control of fermentation conditions can also effectively solve the problem of uncontrolled mono/di-RL ratio in RL products, ensuring a high proportion of a specific RL type. Marco et al. [50] evaluated the effects of different carbon sources (glycerol (2% *v*/*v*), glucose (0.4% *w*/*v*), myristic acid (19.43 mM), and turnip oil (0.4% *v*/*v*)) on the synthesis of mono-RLs and di-RLs in the *P. aeruginosa* PAL 05 isolate. The results showed that mono-RLs dominated the product when hydrophobic carbon sources were provided, while di-RLs prevailed with hydrophilic carbon sources. This conclusion was also validated in Nitschke’s [69] study on reusing waste as carbon sources. In certain special strains, such as *P. aeruginosa* A39-1, glycerol as a carbon source enables the exclusive secretion of mono-RLs [70]. These findings indicate that different carbon sources regulate the expression of the *rhlC* gene, thereby influencing the type of RL product.

Dissolved oxygen content also influences product composition, such that di-RLs dominate under aerobic conditions, while mono-RLs account for a higher proportion under anaerobic conditions. For example, Zhao et al. [71] found that the mono-RL content in RL fermentation broth produced anaerobically (94.7%) was higher than that produced aerobically (54.8%). Transcriptome sequencing revealed that the *rhlC* gene is downregulated under anaerobic conditions, resulting in the reduced conversion of mono-RLs to di-RLs. RL fermentation also varies with temperature; *Pseudomonas* sp. SQ6 undergoes high-temperature fermentation at 40 °C, with mono-RLs accounting for 96.1% of the product [72].

Overall, these studies collectively demonstrate that the substrate and fermentation conditions can modulate the type and composition of RLs during fermentation. This flexibility makes RL production more adaptable to the diverse market demands, endowing it with broad industrial prospects.

### 4.3. Selective Fermentation of Mixed RLs

RL physicochemical properties are structure-dependent. Mixed RLs can integrate the advantages of mono-RLs and di-RLs to a certain extent, providing new ideas for the selective production and application of RLs. Wu et al. [61] constructed *P. aeruginosa* SG*rhlC* by introducing an additional *rhlC* gene into *P. aeruginosa* SG and investigated the relationship between its activity and di-RL proportion. They studied the changes in the physicochemical activities of three RL mixtures with different ratios as the di-RL proportion increased, and found that higher di-RL proportions were associated with lower CMC, better surface activity, higher oil-washing rate, weaker emulsifying activity, and lower antibacterial activity. Such mixed RLs exhibited good surface activity, low CMC, and excellent wetting, foaming, desorption, and dispersion properties [65].

Fermentation of different strains or modified strains enables the production of fermentation broths with the desired di-RL proportions. A typical example is fermenting *P. aeruginosa* WB505 with cooking oil fume condensate (COFC), where the total relative abundances of mono-RLs and di-RLs are 49.7% and 50.3%, respectively [73]. The RLs produced by *P. aeruginosa* SG include 54.76% di-RLs and 45.24% mono-RLs under aerobic conditions, while the mono/di-RL ratio reaches as high as 95:5 under anaerobic conditions [64]. Thus, the mono/di-RL ratio in RL fermentation broth can be artificially controlled by adjusting the oxygen content to obtain the desired product. Similarly, Jiang et al. [74] found that the mono/di-RL ratio in the fermentation broth of *Pseudomonas* sp. CH1 was 37:63 under aerobic conditions and 88:12 under anaerobic conditions.

In addition to oxygen content, different fermentation substrates also lead to variations in product composition. Marco et al. [50] found significant differences in the mono/di-RL ratio when using glycerol, glucose, myristic acid, and Brassica carinata oil as fermentation substrates (Table 2). Even within the same type of carbon source (either hydrophobic or hydrophilic), the compositions of RLs varied considerably. For example, the mono/di-RL ratio was 21:79 with glycerol as the substrate, but 45:55 with glucose. These differences enable the production of RL mixtures with specific ratios. Meanwhile, Santos’s [75] experiments confirmed that this phenomenon is not an exception but universally applicable to various RL-producing strains. In addition to carbon sources, nitrogen sources also affect the product composition during fermentation. When glycerol was used as the carbon source, the mono/di-RL ratios were 67:33 and 54:46 with ammonium sulfate and sodium nitrate as nitrogen sources, respectively. This also provides inspiration and feasibility for artificial regulation. Beyond regulating the RL ratio, it is also necessary to balance yield and performance during fermentation. Among carbon sources such as glycerol, ethanol, soybean oil, and olive oil, ethanol and vegetable oils show good potential for higher yields, but glycerol produces RL mixtures with better performance when paired with selected *P. aeruginosa* strains [75]. Consequently, different carbon–nitrogen source combinations can be explored to study their effects on RL production, ensuring a balanced mono/di-RL ratio while optimizing yield and performance.

## 5. Applications of RLs

### 5.1. Antibacterial Agents

RLs exhibit significant broad-spectrum antibacterial and antibiofilm activities, and their mechanisms of action are closely related to structural specificity, which has emerged as a key focus for their high-value applications. Panatula’s study reported that di-RLs isolated from *Nocardiopsis dassonvillei* B2 exhibits multifunctional bioactivity, including antibacterial and antibiofilm effects as well as anticancer-related outcomes, highlighting the therapeutic promise of specific RL congeners [76]. RLs exhibit significant broad-spectrum antibacterial and antibiofilm activities, and their mechanisms of action are closely related to structural specificity, which has emerged as a key focus for their high-value applications. Ceresa et al. [77] found that RLs at concentrations of 0.06 mg/mL and 0.12 mg/mL significantly inhibited the growth of *Staphylococcus aureus* and dispersed up to 93% of preformed biofilms. Both mono-RLs and di-RLs achieved over 90% inhibition rates against Gram-positive bacteria and fungi. It is generally accepted that the main antibacterial mechanism of RLs is to disrupt cell membrane integrity as they insert into the lipid bilayer to increase permeability and cause leakage of intracellular components; at higher concentrations, they can directly lyse the cell membrane and ultimately induce cell death [78]. Notably, numerous studies have shown that mono-RLs generally exhibit superior antibacterial efficacy compared to di-RLs. For example, Zhao’s [63] experiments showed that mono-RLs produced inhibition zone diameters exceeding 25 mm and inhibition rates above 90% against the tested bacteria and fungi, outperforming di-RLs. In another study by Zhao [65], the half-maximal inhibitory concentration (IC_50_) of mono-RLs was consistently below 5 mg/L, whereas that of di-RLs ranged from 10 to 20 mg/L. This difference in potency is mainly due to structural variations affecting their physicochemical properties. The stronger lipophilicity of mono-RLs enables more effective interaction with the hydrophobic phospholipid bilayer of microbial cell membranes, as they not only insert and disrupt the membrane structure and thus lead to denaturation or lysis, but also penetrate into cells more easily [79]. Once inside the cell, mono-RLs induce a more intense oxidative stress response, resulting in a sharp increase in intracellular reactive oxygen species (ROS) levels. Excessive ROS cause irreversible oxidative damage to nucleic acids, proteins, and bioactive enzymes, thereby disrupting normal cellular growth, metabolism, and respiratory functions, and ultimately leading to cell death [80]. Thus, the combined effects of stronger membrane-targeting disruption and more severe intracellular oxidative damage are the key reasons for the superior antibacterial activity of mono-RLs compared to di-RLs.

Mono-RLs also exhibit good preventive effects against biofilms, which are a major concern in the food processing industry as they cause equipment corrosion and food spoilage. Recent studies reported the robust antibiofilm performance of RLs against Gram-positive biofilms relevant to food safety (*Bacillus cereus*), and synergistic strategies are being explored to improve removal efficiency [81]. In implant-related contexts, Tambone et al. [82] demonstrated that RL 89 (R89BS) effectively counteracts *Streptococcus oralis* biofilm formation on titanium while preserving osteoblast behavior, supporting the potential of RL-based antibiofilm coatings for preventing peri-implant infections. In summary, RLs inhibit biofilms by blocking the initial adhesion stage, specifically through reducing the surface free energy of the membrane, interacting with certain extracellular polymeric substance (EPS) proteins, and inducing EPS loss [83].

Along with it comes the safety issue of RLs, and there are two complementary perspectives on human toxicology, regulatory/standard acute toxicity endpoints, and cell-based cytotoxicity/irritation studies (relevant to therapeutic and topical use). As regulatory context, U.S. EPA documentation for RL biosurfactants reports very low acute toxicity categories (acute dermal LD_50_ > 5000 mg/kg), which we cite and briefly interpret [84]. For structure-resolved cellular responses, many studies that directly compared mono- vs. di-RLs in cytotoxicity assays have discussed that membrane interactions and cytotoxic potency can differ by rhamnose headgroup composition, supporting the need for form-specific safety assessments [85].

### 5.2. MEOR

Microbial Enhanced Oil Recovery (MEOR) is a long-standing technology that has attracted sustained attention for enhancing crude oil recovery during tertiary oil recovery by leveraging microorganisms or their metabolites. Among the various MEOR technologies, the application of biosurfactants is particularly prominent, with RLs receiving significant attention due to their excellent surface activity. Although all RL congeners have MEOR application potential, di-RLs offer greater advantages as they maintain good stability under the variable physicochemical conditions similar to reservoir environments. Studies have shown [86] that di-RLs exhibit optimal micellar solubilization capacity and interfacial properties, along with excellent physicochemical characteristics such as stable emulsifying ability, low oil–water interfacial tension, and good wettability [87]; they also achieve the highest emulsification index (100%) and wettability reversal efficiency (100% at 25 ppm). In addition, di-RLs have a high acute toxicity value (454 mg/L), indicating better environmental compatibility and application safety. These results suggest that product combinations rich in di-RLs are considered to have better application prospects and effects in MEOR.

Beyond enhancing oil recovery, RLs also exhibit excellent performance in oily sludge treatment. The washing process of oily sludge by RLs mainly involves two stages [88]: the first is the desorption of crude oil from the surface of solid particles through wetting and penetration, and the second is the dispersion of crude oil in the aqueous phase to form a stable emulsion by virtue of the RLs’ emulsifying capacity. Zhao et al. [64] found that after treatment at 35 °C and 180 rpm for 24 h, RLs (both at 200 mg/L) produced by the *P. aeruginosa* SGΔ*rhlC* and SG strains desorbed 1.904 g and 2.165 g of crude oil, respectively, from an initial 10 g oily sludge sample (washed with 100 mL rhamnolipid solution). Although the applied concentration was the same, the washing efficiency differed due to structural and property variations in the RLs produced by the two strains.

RLs intended for MEOR must remain functional under reservoir-like high temperature and high salinity/mineralization, including Ca-rich brines that can compromise many anionic surfactants. Importantly, stability can be congener-dependent: mono- and di-RLs differ in headgroup size and aggregation behavior, which affects tolerance to ionic strength and temperature. Reported studies show that both forms can preserve key oil recovery-relevant effects across broad conditions, while congener-resolved comparisons suggest that di-RLs may be more robust than mono-RLs at high ionic strength [89]. Recent assessments under combined stressors of high salinity, elevated temperature and high pressure further validate the applicability of rhamnolipids in harsh reservoir environments while also emphasizing the imperative for standardized assays conducted in realistic formation brines with explicit mono-/di-RL speciation [90]. Recent MEOR/EOR reviews further emphasize that optimal oil displacement and wettability alteration require matching the RL composition and formulation to specific brine chemistries and reservoir conditions [12].

### 5.3. Anticancer Agents

Di-RLs have a unique application potential in antitumor therapy as they can selectively inhibit the proliferation of various cancer cells and exhibit structure-dependent biological activity. Notably, existing studies generally indicate that the anticancer activity of di-RLs is significantly superior to, or even exclusive of, that of mono-RLs, in contrast to the structure–activity relationship observed in their antibacterial activity. Multiple in vitro studies have provided evidence for this. For example, Wang et al. [91] found that di-RLs isolated from *P. aeruginosa* B189 were cytotoxic to human breast cancer MCF-7 cells, with a minimum inhibitory concentration (MIC) of 6.25 μg/mL; under the same conditions, mono-RLs showed no significant activity. The study also observed a key characteristic that at a concentration of 50 μg/mL, di-RLs had no significant effect on the growth of normal monkey kidney (Vero) cells, initially revealing their potential selective toxicity toward cancer cells. Another study by Thanomsub et al. [92] further confirmed the potent and broad-spectrum potential of di-RLs, reporting that di-RLs produced by *P. aeruginosa* M14808 had significant antiproliferative activity against human non-small cell lung cancer H460 cells and breast cancer MCF-7 cells, with an IC_50_ as low as 5 μg/mL and 1 μg/mL, respectively. Similarly, no comparable activity was observed for mono-RLs or their crude extracts in this study. More recently, a di-RLs from *Nocardiopsis dassonvillei* B2 was reported to exhibit multifunctional bioactivity including anticancer, antibacterial, and antibiofilm effects, further broadening biomedical application prospects beyond Pseudomonas-derived products [74].

Unfortunately, the specific molecular mechanisms by which di-RLs inhibit cancer cells have not been fully elucidated. Current speculation involves multiple pathways, such as inducing apoptosis, arresting the cell cycle, or disrupting cell membrane signaling [93], and the mechanism underlying their excellent selective toxicity is a key focus of future research. In summary, existing evidence highlights the value of di-RLs as structure-specific anticancer lead molecules. However, to develop them into genuine therapeutic agents, more in-depth and systematic research on their mechanisms of action, in vivo efficacy, and safety is urgently required.

### 5.4. Bioremediation

RLs have very promising application prospects and excellent biosafety. We summarize evidence that RLs are generally readily biodegradable and that aquatic ecotoxicity varies with structure and test organism. For example, Zhao et al. evaluated purified mono-RLs and reported EC_50_ values in the “slightly toxic” range for aquatic tests, providing structure-resolved information for the mono-RL class [94]. As a category of biosurfactants, the applicability of RLs in soil and sediment remediation is attributed to their direct solubilization function and the capacity to indirectly promote biodegradation via adjusting the surface characteristics of microbial cells [95]. Studies have further revealed the differential impacts of RLs with different structures on the migration and transformation of pollutants in complex environmental media. Taking the behavior of triclosan (TCS), a typical hydrophobic organic pollutant, in the sediment–water system as an example, both mono-RLs and di-RLs can effectively promote the desorption and solubilization of TCS, but their efficiencies differ significantly. Equilibrium experiments showed that di-RLs outperformed mono-RLs in both molar solubilization ratio and micelle/aqueous phase partition coefficient for TCS (0.35 > 0.254, 645 > 391.3), indicating that di-RLs have a stronger solubilization-enhancing effect on TCS than mono-RLs. The environmental behavior of both is highly correlated with sediment clay content. As the RL concentration increases (0.05–7.5 mM), the apparent partition coefficient of triclosan continuously decreases, indicating that more pollutants are transferred from the solid phase to the aqueous phase. Notably, at higher concentrations (>2.5 mM), di-RLs exhibit stronger partition regulation capacity, driving more TCS to partition into the aqueous phase [96]. In terms of desorption kinetics, both RL types can effectively promote the release of TCS from sediments, but di-RLs have higher desorption efficiency. At a concentration of 5 mM, the desorption percentage of TCS by di-RLs can be 1.8–2.4 times that of mono-RLs [97]. This performance difference is attributable to their distinct micellization behaviors and aggregation structures, as di-RLs tend to form smaller micelles with larger specific surface area and thus possess better mass transfer efficiency and kinetic advantages in competing for sediment adsorption sites and solubilizing pollutant molecules. In contrast, mono-RLs are more prone to forming vesicles or larger lamellar aggregates, with their solubilization sites possibly confined within the aggregates, leading to relatively slow mass transfer and thus lower desorption efficiency. This clearly indicates that in environmental remediation applications, selecting or tailoring RLs with appropriate structures such as high di-RL ratio based on specific pollutants and soil properties is a key strategy to optimize remediation efficiency.

### 5.5. Agricultural Applications

Beyond industrial and biomedical uses, RLs are increasingly explored in agriculture as bioprotectants and biostimulants. In crop protection, RLs can provide a dual mode of action by combining direct antimicrobial effects with the ability to elicit plant immunity, thereby improving resistance against phytopathogens. A focused mini-review summarizes evidence that RLs can inhibit plant pathogens and trigger defense responses, positioning them as promising tools for more sustainable plant protection strategies [98]. Experimental studies further demonstrate that RLs from *Pseudomonas aeruginosa* can protect Brassica napus tissues against Botrytis cinerea via a combination of defense activation and direct antifungal activity [99]. In addition, formulation studies (RL-stabilized nanoemulsions) show enhanced delivery and stronger immune-inducing performance without apparent detrimental effects in the tested plant systems, supporting practical deployment concepts for field applications [100]. Parallel to plant protection, RLs have been proposed as biostimulants, with recent reviews highlighting their potential roles in improving soil/plant performance and tolerance to abiotic stresses [101]. Nevertheless, much of the agricultural literature evaluates unresolved rhamnolipid mixtures or formulated products, and congener-resolved comparisons (mono- vs. di-RLs) for efficacy thresholds, phytotoxicity windows, and non-target safety remain limited—representing an important gap for future translation.

## 6. Discussion

This review summarizes the differences between mono-RLs and di-RLs in structure, properties, and applications. Due to the difference in the number of rhamnose groups, the two exhibit distinct differentiation in interfacial properties and functions. Mono-RLs are highly hydrophobic with excellent surface activity and a prominent solubilization capacity for hydrophobic pollutants, making them suitable for daily chemicals, environmental remediation, and antibacterial applications. In contrast, di-RLs possess superior interfacial activity, larger hydrophilic heads, and high hydrophilic–lipophilic balance values, enabling them to form stable emulsions and foams, resist hard water, and exhibit specific biological activities, which makes them ideal for microbial oil recovery, nanoformulations, and biomedicine. This “structure–property–function” relationship provides a basis for targeted applications. Currently, tailor-made synthesis has become the core strategy for precisely guiding metabolic pathways to achieve controllable ratios of target products by regulating genes such as *rhlB* and *rhlC* and optimizing fermentation conditions such as carbon source nature and dissolved oxygen, thus marking a new phase of demand-driven design in rhamnolipid production.

As a microbial-derived biosurfactant with significant application potential, the in-depth research and industrialization of RLs still face a series of in-depth scientific challenges and strategic opportunities. On the production technology front, achieving efficient and economical large-scale manufacturing is the core bottleneck for industrial transformation, and the solution relies on the in-depth integration of synthetic biology and fermentation engineering. On the one hand, it is necessary to rationally design and reconstruct RL synthesis pathways in non-pathogenic and efficient heterologous hosts and maximizing metabolic flux through strategies such as dynamic regulation and cofactor engineering. On the other hand, it is essential to simultaneously develop low-cost fermentation processes using agricultural and industrial by-products or non-food biomass as raw materials, coupled with efficient and green downstream extraction and purification technologies such as membrane separation and foam fractionation to overcome cost and environmental constraints.

In terms of application expansion, there is a need to go beyond traditional fields such as environmental remediation and oil recovery. Research should explore RLs’ potential as templates or stabilizers in green nanomaterials synthesis, the mechanism of RLs acting as plant biostimulants or rhizosphere immune inducers in sustainable agriculture, and the feasibility of RLs as solubilizers or dispersants in the migration, transformation, and remediation of emerging pollutants. Collectively, this will facilitate the translation of RLs, an extremely promising class of biomolecules, from laboratory-scale research to large-scale commercial applications.

## Figures and Tables

**Table 2:** Influence of different fermentation conditions on RL types.

Strain	Condition	Mono/Di-RL Ratio	Reference
*P. aeruginosa* L05 (Different Carbon Sources)	Glycerol	21:79	[50]
	Glucose	45:55	
	Myristic Acid	56:44	
	Brassica Carinata Oil	92:8	
*P. aeruginosa* SG (Different Oxygen Levels)	Aerobic	45:55	[71]
	Anaerobic	95:5	
*Pseudomonas* sp. CH1 (Different Oxygen Levels)	Aerobic	37:63	[73]
	Anaerobic	88:12	
*P. aeruginosa* PA1 (17) (Different Carbon Sources)	Glycerol	15:85	[75]
	Soybean Oil	67:33	
	Ammonium Sulfate	67:33	
	Sodium Nitrate	54:46	

## Data Availability

No new data were created or analyzed in this study. Data sharing is not applicable to this article.

## Abbreviations

The following abbreviations are used in this manuscript:

RLs	Rhamnolipids
mono-RLs	Mono-rhamnolipids
di-RLs	Di-rhamnolipids
HLB	Hydrophile–lipophile balance
O/W	Oil-in-water
W/O	Water-in-oil
CMC	Critical micelle concentration
MEOR	Microbial enhanced oil recovery
SDS	Sodium dodecyl sulfate
AES	Alcohol ether sulfate
APG	Alkyl polyglycoside
ACS	Cocamidopropyl sulfate
SF	Silk fibroin
ROS	Reactive oxygen species
EPS	Extracellular polymeric substance
TCS	Triclosan
